# Four successful pregnancies in a woman after Fontan palliation: a case report

**DOI:** 10.1186/1752-1947-8-350

**Published:** 2014-10-21

**Authors:** Rym Gribaa, Mehdi Slim, Sana Ouali, Elies Neffati, Fehmi Remadi, Essia Boughzela

**Affiliations:** 1 Department of Cardiology, Sahloul University Hospital, Sousse, Tunisia

**Keywords:** Fontan circulation, Grown-up congenital heart disease, Pregnancy

## Abstract

**Introduction:**

A Fontan operation is performed to provide palliation for patients with many forms of highly complex congenital heart disease that cannot support a biventricular circulation. Increasing numbers of women who have undergone these connections in childhood are now reaching their childbearing years, and some are becoming pregnant. The low flow and fixed cardiac output of a Fontan circulation poses several problems during pregnancy.

**Case presentation:**

We report the case of four successful pregnancies in a 31-year-old Tunisian woman with congenital tricuspid atresia after Fontan operation. Her pregnancies resulted in delivery of four healthy neonates. Her clinical status remained unchanged.

**Conclusions:**

This case suggests that patients after adequate Fontan palliation could complete pregnancy without long-term cardiac sequelae. Intensive care should be provided with specialists, including a neonatologist, anesthesiologist and cardiologist.

## Introduction

The Fontan operation was introduced as a palliative procedure for patients with tricuspid atresia and has since been used extensively to provide palliation for patients with several forms of congenital heart disease that cannot support biventricular circulation. The vena cava flow is directed to the pulmonary arteries without passing through the ventricle. It was initially described in 1971 [1]. Since then, the Fontan procedure has evolved over the years, most notably with the substitution of right atrial-to-pulmonary artery anastomosis with cavopulmonary connections [2]. This progress resulted in significantly improved late outcomes; an increasing number of women who have undergone these connections in childhood are now surviving into adulthood, and some of them are becoming pregnant. Pregnancy after Fontan procedure has one of the highest risks in women with congenital heart disease and therefore requires careful prior consideration. Strict perinatal care is mandatory in these women. Reports on pregnancy in women with a prior Fontan connection are scarce. Here we describe four successful pregnancies in a woman after Fontan palliation.

## Case presentation

We report the case of a 31-year-old Tunisian woman with a past medical history of congenital tricuspid atresia and pulmonary stenosis. In 1987, she underwent Blalock–Taussig operation at the age of 5 years and Fontan operation at the age of 12. The procedure included lateral atrial tunnel combined with bidirectional cavopulmonary anastomosis.

**Figure 1:**
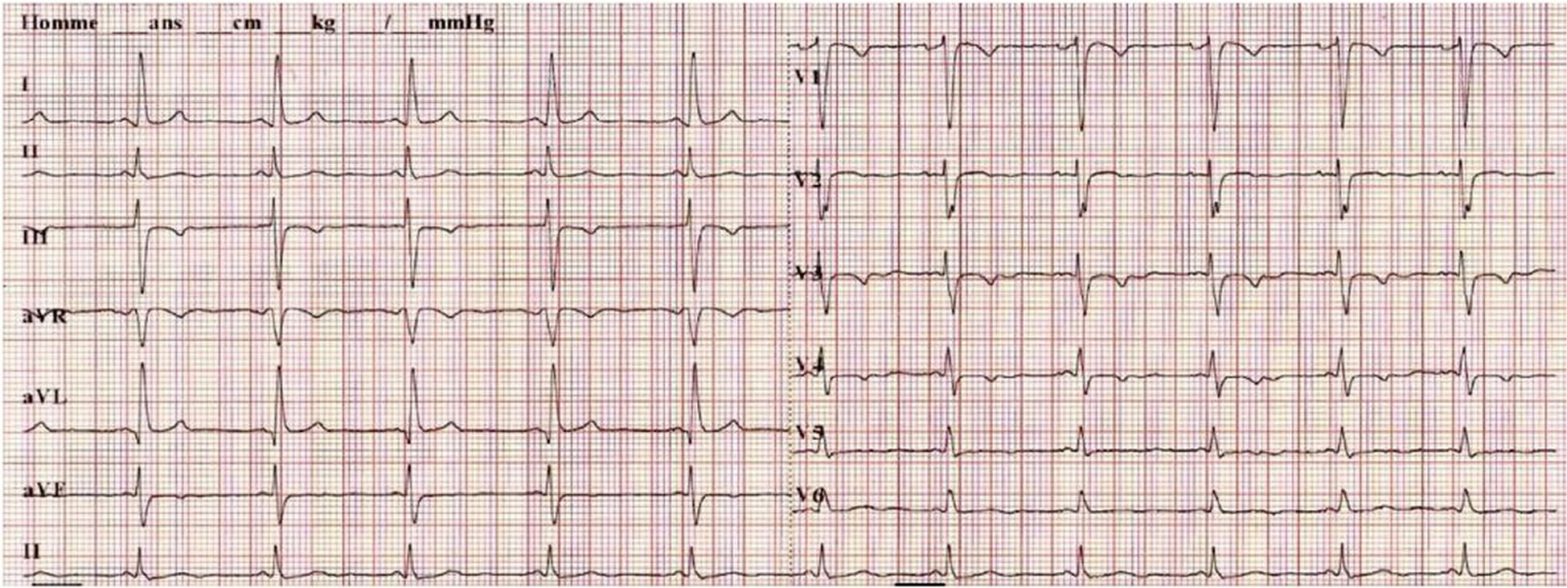
**Electrocardiogram.** The electrocardiogram shows sinus rhythm with incomplete left bundle branch block.

**Figure 2:**
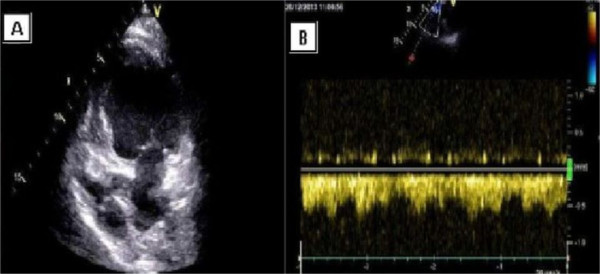
**Transthoracic echocardiography.** Panel **A**: Apical four chamber view showing tricuspid atresia. Panel **B**: Doppler interrogation of the anastomosis (superior vena cava to right pulmonary artery) revealed a laminar pattern.

Recovery after treatment was uneventful. After surgery the patient was in New York Heart Association (NYHA) class I functional status. She was on vitamin K antagonists (acenocoumarol) for only 7 years: she withdrew from them herself. Contraception was advised and programmed pregnancy was recommended; however, she consulted when she was already pregnant. There is no ethical issue about medical termination of pregnancy in Tunisia. A summary of her four pregnancies is given in Table 1. She refused tubal ligation after her first pregnancy. Her clinical status was satisfactory during pregnancy. The pregnancies did neither cause any further limitation of her exercise capacity (NYHA I) nor episodes of supraventricular arrhythmia or thrombosis complications. The puerperia were uneventful without obstetric complications, especially postpartum hemorrhage. Fetal echocardiography, performed regularly during her four pregnancies, showed no congenital defects and no other abnormalities.Currently the patient is in a good general condition and a NYHA functional class I. A physical examination revealed a regular heart rate of 75 beats per minute, oxygen saturation of 96%, and no murmur nor signs of congestive cardiac failure. The 6-minute walk test showed good exercise tolerance. The findings of the electrocardiograms were sinus rhythm with incomplete left bundle branch block (Figure 1). Her echocardiography showed a preserved left ventricle ejection fraction of 69%, without any significant atrioventricular regurgitation. Both atriums communicate by a large atrial septal defect. Doppler interrogation of the anastomosis revealed a laminar pattern (Figure 2). Her four children develop well and no abnormalities of their cardiovascular system were detected.

**Table 1:** Summary of the four pregnancies undergone by the patient

**Year**	**Age (years)**	**Complications during pregnancy**	**Term of delivery (weeks)**	**Route of delivery**	**Baby sex**	**Baby status after birth**	**Birth weight (g)**	**Complication after delivery**
1993	21	None	38	Vaginal	Female	Good	2900	None
1998	26	None	37	Vaginal	Female	Good	2500	None
1999	27	Premature rupture of membranes	32	Vaginal	Male	Good	2100	None
2002	30	None	38	Vaginal	Female	Good	2600	None

## Discussion

In normal pregnancy with an increase in plasma volume, cardiac output continues to increase up to mid-pregnancy (30 to 40% above non-pregnant levels) as does circulating volume (up to 50%) and heart rate (up to 25%). For post-Fontan patients, the ability of the right atrium and functioning single ventricle to generate and tolerate the normal cardiovascular adjustment to pregnancy holds the key to successful pregnancy [3]. Hemodynamic changes observed during pregnancy especially increased heart rate, stroke volume and cardiac output with parallel reduction of vascular resistance are particularly disadvantageous in women after Fontan operation, due to their significant dependence on venous return and limited capability to increase the stroke volume. Such patients during pregnancy are at risk of heart failure, arrhythmias, ascites and even death caused by elevation of central venous pressure. Cardiac assessment during pregnancy should be made by echocardiography, electrocardiogram and brain natriuretic peptide [4]. They are also at risk of thrombosis and stroke. In fact, most patients after Fontan palliation need warfarin to prevent thrombosis in their surgically altered low flow circulation, and it should be substituted for unfractionated or low-molecular-weight heparin during pregnancy, especially during early and term pregnancy to prevent teratogenesis and to avoid excessive bleeding at labor due to warfarin [5]. Fetal complications were also observed. These complications were caused by the fixed low cardiac output that accompanies Fontan circulation. In fact low blood flow to the uterus and placenta could result in fetal growth restriction, non-reassuring fetal status and intolerance of labor due to metabolic disturbance [3]. Despite these complications, several small case series have been reported in the literature with generally favorable results. To the best of our knowledge, this is the only case of four successful pregnancies in a woman after Fontan palliation resulting in normal delivery of four healthy neonates without significant complications. A summary of different case reports on pregnancy in women after Fontan procedure found in the literature is given in Table 2.

**Table 2:** Overview of the literature on pregnancy after Fontan repair

**Authors (reference)**	**Patients (number)**	**Completed pregnancies**	**Miscarriage/abortion**	**Cardiac complications**	**Pregnancy complications**	**Obstetric complications**	**Neonatal complications**
Girod *et al*. [6]	1	1	–	–	–	PL (1)	PD (1), SGA (1)
Hess *et al*. [7]	1	1	–	SVT (1)	–	PL (1)	PD (1), ND (1)
Carmona *et al*. [8]	1	1	–	–	–	–	–
Gerardin *et al*. [9]	1	1	–	SVT (1), HF (1)	PIH (1)	PL (1), F (1)	PD (1)
Carp *et al*. [10]	2	2	1/–	AFL/AF (1)	–	CS (1), PROM (1), PPH (1)	PD (2), SGA (1)
Cohen and Mulvein [11]	1	1	–	AFL/AF (1)	–	PROM (1), PL (1)	PD (1), ND (1)
Osmers *et al*. [12]	1	1	–	–	VAG (1)	CS (1)	PD (1)
Lao *et al*. [13]	1	1	–	–	VAG (1)	PROM (1), PL (1), PPH (1)	PD (1)
Canobbio *et al*. [3]	21	15	13/5	SVT (1), HF (1), NYHA↓ (1)	–	CS (11), PROM (1), PL (1)	CHD (1), PD (1)
Grunwald *et al*. [14]	1	1	–	–	–	PPH (1)	SGA (1)
Hoare and Radford [15]	3	4	5/2	AFL/AF (2), NYHA↓ (1)	–	CS (2), PROM (1), PL (1)	PD (4)
Siu *et al*. [16]	5	5	–	AFL/AF (2), NYHA↓ (2)	–	–	–
Ito *et al*. [17]	1	1	–	–	–	CS (1)	SGA (1)
Drenthen *et al*. [18]	38	4	5/1	AFL/AF (1), NYHA↓ (2)	VAG (1), PIH (1)	CS (3), F (1), PL (1), PROM (1), PPH (1)	PD (2), SGA (2), ND (1)
Overall	78	39	24/8	AFL/AF (7), NYHA↓ (6), SVT (3) HF (2)	VAG (3), PIH (2)	CS (19), PL (8), PROM (6), PPH (4), F (2)	PD (15), SGA (5), ND (3), CHD (1)

AF, atrial fibrillation; AFL, atrial flutter; CHD, congenital heart disease; CS, caesarean section; F, forceps delivery; HF, heart failure; ND, neonatal death; NYHA↓, New York Heart Association class deterioration; PD, premature delivery; PIH, pregnancy-induced hypertension; PL, premature labor; PPH, postpartum hemorrhage; PROM, premature rupture of membranes; SGA, small for gestational age; SVT, supraventricular tachycardia; VAG, vaginal bleeding.

Because of the potential risk of complications of the Fontan pregnancy, delivery and peripartum management should be performed in a center with significant experience in the medical, obstetric, and anesthesiologic care of these patients. However, further studies are needed to determine the effect of the pregnancy on the survival of such patients.

## Conclusions

Women can successfully complete pregnancy after adequate Fontan palliation without important long-term sequelae. Larger prospective studies or registries are needed to assess the outcome of pregnancies in patients with congenital heart disease, especially those with complex anomalies and Fontan palliation.

## Consent

Written informed consent was obtained from the patient for publication of this case report and any accompanying images. A copy of the written consent is available for review by the Editor-in-Chief of this journal.

## Abbreviations

NYHA: New York Heart Association.

## Competing interests

The authors declare that they have no competing interests.

## Authors’ contributions

RG drafted the manuscript. MS contributed in collecting of data. OS contributed in writing the manuscript. EN contributed in correcting the manuscript. FR contributed in correcting the manuscript. EB contributed in analysis, in interpretation of data, in the writing of the manuscript and in the decision to submit the manuscript for publication. All authors read and approved the final manuscript.
